# Factors influencing cardiovascular disease screening uptake and implementation strategies to enhance cardiovascular disease screening uptake in Singapore adults: a multi-method study protocol

**DOI:** 10.3389/frhs.2025.1677666

**Published:** 2026-01-07

**Authors:** Ngoc Huong Lien Ha, Gigi Toh, Mary Ng, Jumana Hashim, Yen-Ting Tina Chen, Shao Chuen Tong, Joyce Tan, Zi Xuen Wong, Pei Fen Sam, Shir Gi Toh, Jun Hui Tan, Ke Xin Eh, Wei Lin Ng, Adelene Ong, Zhen En Ang, Catherine Koh, Zheng Jye Ling, Joanne Yap, Nick Sevdalis

**Affiliations:** 1 Centre for Behavioural and Implementation Science Interventions, Yong Loo Lin School of Medicine, National University of Singapore, Singapore, Singapore; 2 Regional Health System Office, National University Health System, Singapore, Singapore

**Keywords:** cardiovascular disease, health screening, multi-method, co-design, implementation science

## Abstract

**Introduction:**

The prevalence of cardiovascular diseases (CVD) and CVD risk factors such as Type 2 Diabetes Mellitus (T2DM), hypertension and hypercholesterolemia has increased steadily worldwide. Population health screening is a common effort that promotes early detection, better prognosis and reduces disease burden. However, despite nationwide efforts, screening uptake for CVD risk factors in Singapore has remained moderately low (60.2%). Profiles of individuals who do not screen remain largely unknown, making them harder to reach via mainstream screening efforts. Existing literature has yet to organise factors systematically influencing CVD screening uptake, making it difficult to select a set of robust strategies to promote CVD screening uptake. This study aims to identify determinants of screening uptake for T2DM, hypertension and hypercholesterolemia in eligible adults residing in Western Singapore, and develop an implementation strategy toolkit to enhance screening uptake in this population.

**Methods:**

Prospective, theory-informed, two-phased, multi-method study design. *Phase 1*: rapid umbrella review, document review and qualitative interviews (*n* = 20–40) to examine existing evidence about behavioural factors influencing CVD risk factors and strategies implemented to increase uptake. *Phase 2*: identified determinants mapped onto strategies, its feasibility and acceptability. Strategies ranked high will be specified to clarify who will implement them, what actions are required and how they will be implemented in specific settings. The strategies are organised into an actionable toolkit, where the Implementation Research Logic Model technique will be adopted to articulate the interrelationships between determinants, hypothesised causal mechanisms and strategies. Both phases will be guided by established implementation science frameworks and co-design approach.

**Clinical Trial Registration:**

identifier [CRD42024566701].

## 1 Introduction

Cardiovascular diseases (CVDs) are the leading causes of mortality in the world. Globally, CVD-related deaths are projected to be 35% of all recorded deaths in 2030 (1, 2). CVD has been described as a continuum that begins with the presence of cardiovascular risk factors and proceeds via progressive vascular disease to target organ damage, end-organ failure, and death (3). Many cardiovascular risk factors share the same aetiology; therefore, it is essential to identify and treat a patient's overall cardiovascular risk rather than to consider the factors in isolation. Modifiable risk factors for CVD include hypertension, hypercholesterolemia, type 2 diabetes mellitus (T2DM), smoking, obesity, as well as lack of physical activity and stress. In particular, T2DM, hypercholesterolemia and hypertension are three chronic conditions established to be major risk factors for cardiovascular diseases. Hypertension and T2DM have been identified as among the most preventable causes of premature death, while hypertension and hypercholesterolemia are among the top three causes of death in industrialised countries, with similar trends emerging in economically developing countries (4–8).

A key challenge of CVD and CVD risk factor control is the early identification of individuals who are at high risk (9). The chronic nature of these conditions means that many individuals remain undiagnosed until the development of late and more severe complications (10). The proportion of undiagnosed cases from the total disease burden are estimated to be 46.8% for T2DM and 48.3% for hypertension (11, 12). Globally, population-level screening is a widely implemented prevention effort used to detect CVD risk factors (13). Population-level health screening leads to higher identification rates of individuals at risk of CVD (13). However, many patients who are at high risk of CVD or those with established coronary diseases or strokes remain unidentified and inadequately treated (14–16). Despite efforts to advocate systematic screening, a significant proportion of higher-risk individuals do not participate in health screening and, thus, remain undiagnosed (10).

CVDs are the leading cause of disability and mortality in Singapore (10). Since 2017, the Ministry of Health offers a national screening programme named “Screen for Life” to all citizens aged 40 years and above to promote regular health screening. At 5 Singapore Dollar (SGD) (approximately USD3.70 at the time of writing) or less, eligible adults can attend CVD screening at any participating primary care clinics island wide. The latest national preventive health programme called “HealthierSG” (HSG) since 2023 also aims to facilitate screening uptake, for example, citizens are provided with SGD20 Healthpoints upon the first consultation with a family doctor, and access to more affordable medications from HSG-enrolled GP clinics (17). Furthermore, health screening is also organised at the workplaces: several large employers in Singapore encourage their employees to go for regular health screening and/or include health screening in compensation packages (18).

Despite these efforts, the age-standardised proportion of eligible Singaporeans with no previous CVD diagnosis self-reported to have screened for these conditions as per national guidelines remains relatively low (60.2%) (10). There have been attempts to study factors that influence CVD screening participation in the local context (19–21), with findings suggesting demographic variables, health attitudes and logistical factors, like accessibility and time, to be among the determinants of uptake (22–24). Systematic evidence on effective, context-specific, localised interventions to increase screening uptake has yet to be established.

The multi-phased study outlined in this protocol aims to start addressing this gap. As population health screening is a complex, multi-faceted health behaviour, we hypothesise that the use of implementation science theories and concepts could offer an evidence-based structure to systematically understand screening behaviour and its determinants in a specific setting, such as Western Singapore. Implementation dimensions and factors derived from implementation science theories can also contribute to designing tailored, context-driven strategies to enhance CVD screening. Specifically, there is supportive evidence for the use of implementation strategies to improve intervention adoption, implementation, sustainment and scale-up (25). Implementation strategies, defined as interventions that facilitate the uptake of evidence-based interventions (26) can vary in complexity, scale and number of components—from single, discrete strategies, to multi-faceted or multi-component strategies. The use of implementation strategies tailored to address identified barriers was found to be more likely to improve professional practices than not using such strategies (27). In focusing on the identification of relevant implementation strategies, we are cognisant of the critique that there is often little use of theory or framework to guide the process of strategy identification and selection, and a lack of explicit articulation of causal mechanisms between the behavioural or contextual determinants and selected strategies (28–30). Therefore, in this study, implementation science theories and frameworks will be used to systematically develop implementation strategies to improve CVD screening uptake.

### 1.1 Study aims and objectives

The study aims, firstly, to gain a holistic understanding of the behavioural and contextual determinants that affect the screening uptake of CVD risk factors in eligible adults in Western Singapore. Secondly, through the application of implementation science theories and frameworks, stakeholder engagement and co-design methods, the study seeks to design a tailored implementation and behavioural strategy toolkit to enhance CVD risk screening uptake. Our specific objectives are:

1. To identify barriers and facilitators that influence the uptake of screening for T2DM, hypertension and hypercholesterolemia in eligible adults residing in Western Singapore.
2. To develop an implementation and behavioural strategy toolkit to enhance the screening uptake of T2DM, hypertension and hypercholesterolemia in the above population.

## 2 Methods

### 2.1 Study overview: methodological approach and study phases

**Figure 1:**
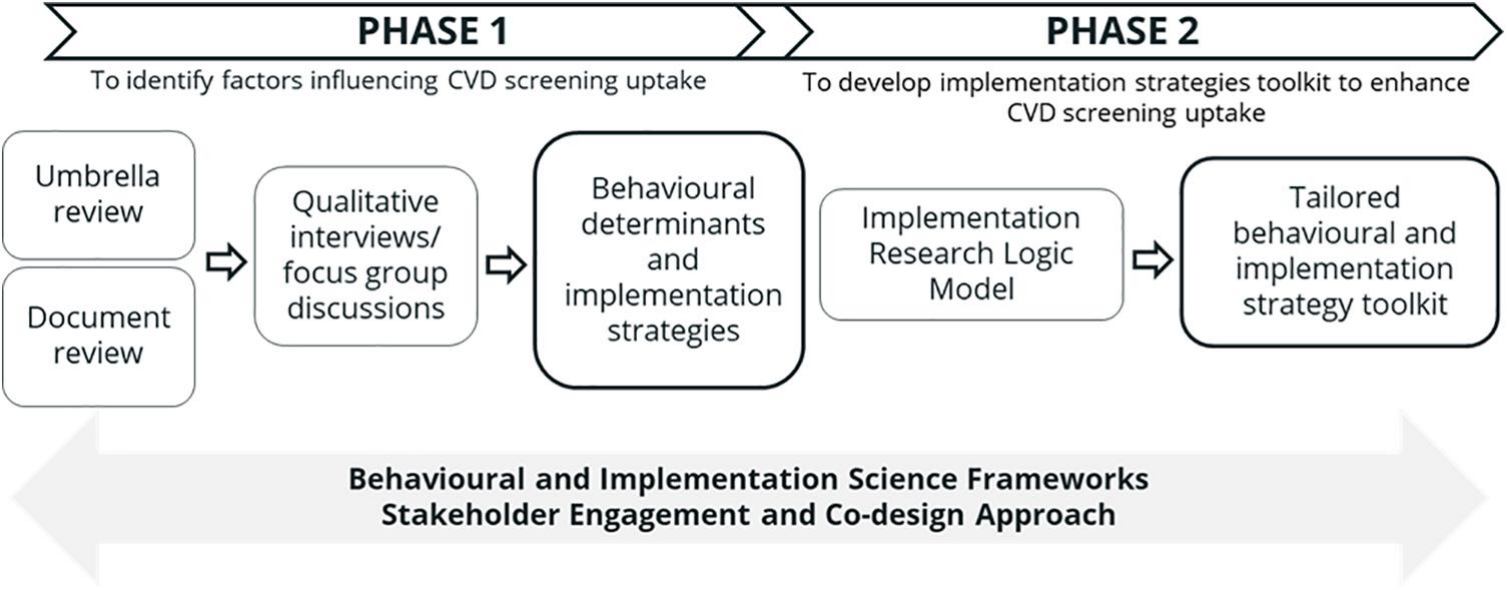
Overall methods summarised across the study's timeline.

The research adopts a prospective, theory-informed, two-phased, multi-methods study design, as outlined in Table 1. Phase 1 uses a rapid umbrella review, document review, and qualitative interviews to explore existing international and national evidence about the barriers and drivers (i.e., determinants) that affect CVD screening uptake, as well as strategies that have been implemented to increase uptake. Phase 2 maps the previously identified determinants to strategies using an approach informed by co-design principles, and articulates the interrelationships between the determinants, hypothesised causal mechanisms and strategies using an Implementation Research Logic Model. Both phases of the study will be guided by relevant implementation science frameworks. The overall study methodology is illustrated in Figure 1.

**Table 1:** The study's overall objectives and methodology.

Objectives	Specific research goals	Methods
1. To identify behavioural barriers and facilitators that influence the uptake of screening for T2DM, hypertension and hypercholesterolemia	To describe factors that affect an eligible individual's participation in screening for T2DM, hypertension and hypercholesterolemia as reported by international literature and local evidence To examine existing implementation and behavioural strategies to enhance participation in screening for T2DM, hypertension and hypercholesterolemia and their effectiveness To understand the experience of primary care providers and residents in providing, implementing and utilising screening programmes for T2DM, hypertension and hypercholesterolemia To explore existing behavioural determinants and implementation strategies from the perspective of key providers, implementers and residents	Rapid umbrella review Document review Qualitative interviews/focus group discussions
2. To develop an implementation and behavioural strategy toolkit to enhance the screening uptake of T2DM, hypertension and hypercholesterolemia in the Western region of Singapore	To co-design with stakeholders a list of selected implementation and behavioural strategies that are evidence-informed, feasible, acceptable and implementable To articulate the working mechanism of selected implementation or behavioural strategies	Implementation Research Logic Model Implementation Mapping

### 2.2 Stakeholder engagement and co-design approach

Stakeholder engagement has been increasingly promoted as an important pathway to achieving impact in implementation research (31). By embedding stakeholder engagement in the research process, the study aims to generate shared commitment and build flexibility to accommodate inputs from stakeholders (31). This approach will also contextualise knowledge and evidence from the literature (which we anticipate to stem largely from Anglo-Saxon countries/cultures), as well as minimise the influence of researchers’ personal biases in the research process.

**Figure 2:**
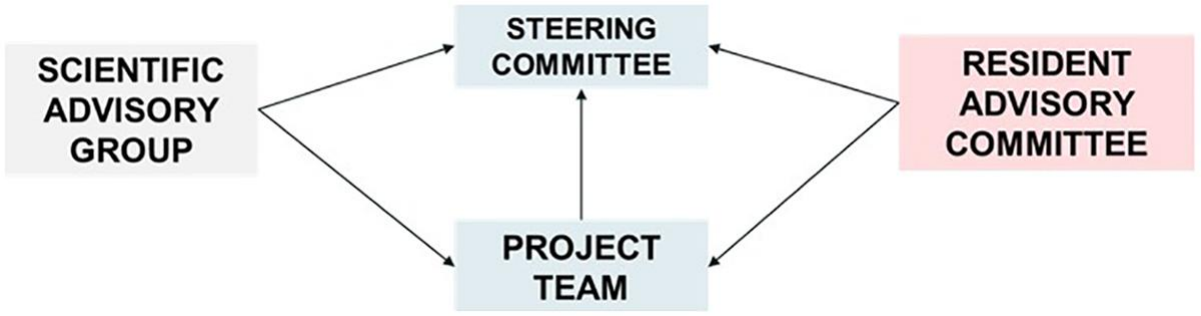
The study's overall governance structure.

The major stakeholders in the context of the study include local residents who are the target population for the CVD screening programme and the Regional Health Systems Office (RHSO: funder and policy maker in charge of the CVD screening programme, among other programmes, reporting directly to the Ministry of Health). Jointly with the RHSO, the research team designed a governance framework for the study to facilitate stakeholder engagement (Figure 2) (31). The study Steering Committee, which comprises the study leads and the RHSO leadership team, collaborates to steer the study as carried out by the Project Team, which consists of scientists and RHSO staff. Following internal discussions on how best to mobilise residents into this framework, the decision was made to establish a separate Resident Advisory Committee (RAC) to ensure that residents would have protected time and space to express their views as the research evolved. The RAC will aim to leverage residents’ insights to enhance the study's relevance and impact. The study team will work with the RHSO partners to identify and recruit eligible residents aged 40 and above living in Singapore's Western region who are keen to share their views or experiences of health screening. Identification and involvement of RAC members will be an iterative and ongoing process, and we envisage that the RAC will participate in quarterly meetings. We intend to explore their experiences of contributing to a research study like the one reported here and offer them capacity development activities as required. The stakeholder framework will be completed through a Scientific Advisory Group, comprising implementation and health service researchers and primary care academic clinicians.

At the time of development of this research protocol, the shared view of the research and RHSO teams (i.e., of the study Steering Committee) is that the optimal design of the overall stakeholder engagement framework remains unknown. As such, the proposed arrangement represents a “study within the study”: the research team keep an auto-ethnographic diary of the stakeholder engagement framework, which will be published as a key study output. This will help us better understand the level, outputs and challenges of recruiting, preparing and achieving stakeholder engagement within the Singapore context—a setting in which, to our knowledge, very few studies have reported such approaches and their effects.

## 3 Study phase 1: explore factors that influence the uptake of screening for T2DM, hypertension and hypercholesterolemia

### 3.1 Rapid umbrella review

#### 3.1.1 Aim

This review aims to identify the barriers and facilitators (i.e., the determinants) that influence screening uptake for T2DM, hypertension and hypercholesterolemia in the general population, and to assess existing implementation strategies and their effectiveness to enhance the screening uptake of these CVD risk factors. The research questions of the review are:

1. What are the behavioural determinants that affect an individual's participation in screening for CVD risk factors?
  a. What are the common determinants for T2DM, hypertension and hypercholesterolemia?
  b. What are the unique determinants for each of the three conditions respectively?
2. What are the existing implementation strategies and their effectiveness to increase screening participation for CVD risk factors?
  a. What are the common strategies for T2DM, hypertension, and hypercholesterolemia?
  b. What are the unique strategies for each of the three diseases respectively?
3. What are the characteristics of the general population who typically do not participate in screening?

The methodology for the review will be guided by the well-established Joanna Briggs Institute umbrella review method (32), which comprehensively and systematically synthesises evidence from systematic reviews and meta-analyses. The findings will be reported following the Preferred Reporting Items for Systematic Reviews and Meta-Analyses (PRISMA) checklist for Scoping Reviews guidelines. The protocol of the review was registered on PROSPERO (PROSPERO registration number: CRD42024566701) on 15 July 2024.

#### 3.1.2 Search strategy

Search terms will be developed based on five concepts: (1) general population without prior diagnosis of selected CVD, (2) population-level health screening, (3) determinants and implementation strategies, (4) uptake in screening and (5) reviews. Appendix 1 presents the full search strategy. The searches will be conducted on three databases: PubMed, Embase and Ovid PsycInfo, as they are the most common databases for behavioural sciences that will result in the retrieval of the highest number of unique references (33).

#### 3.1.3 Eligibility criteria

The eligibility criteria are developed based on the Population, Intervention, Comparison, Outcomes and Study Design (PICO) framework (Table 2). To improve the timeliness of a rapid review, searches are limited to reviews published between 2014 and 24, in English. Manual search in the references of the articles retained in the umbrella review will also be conducted to detect any relevant article not retrieved with the electronic search. Appendix 2 presents the full eligibility criteria for article inclusion.

**Table 2:** The rapid umbrella review's search strategy using the PICO framework.

Component	Description
Population	General population who are above 40 years old and do not have prior diagnosis of T2DM, hypertension and hypercholesterolemia. Prior diagnosis can be described as self-reports or objective assessments in the reviews or studies. Healthcare providers (such as primary care physicians, nurses and other allied health staff), if the population who receives screening fits previous criteria.
Intervention	Factors, determinants or predictors that influence screening uptake, adherence or participation. Behavioural or implementation strategies aimed to increase screening uptake, adherence, or participation of population-level health screening for CVD and risk factors (i.e., at least one of the three CVDs). Strategies will have been tested, assessed directly, inferred, or evaluated.
Comparator	Not applicable. Review articles consisting of studies without comparator groups are included in the review.
Outcome	Data on uptake, adherence, or participation rates of screening for CVD and risk factors.

#### 3.1.4 Data collection

##### 3.1.4.1 Study selection

The study selection stage is an iterative process that involves searching the literature, refining the search strategy and reviewing articles for study inclusion. Sources of data that describe a structured review of evidence and/or provide summaries or recommendations based on evidence are included. This will include systematic reviews, meta-analyses, scoping reviews, short or rapid reviews, practice guidelines, clinical guidelines, and review reports/working papers.

##### 3.1.4.2 Data extraction form

The study team will design a data collection form and pilot it using data extracted from a small sample of reviews (*n* = 5). Extracted data from two reviewers will be compared and discrepancies will be resolved via team consensus. Subsequent modifications may be made to the data extraction form where required.

##### 3.1.4.3 Title and abstract screening

Articles that fit the eligibility criteria will be retrieved into EndNote 21 and de-duplicated. Three study team members (HNHL, GT, MN) will independently screen 10% of titles and abstracts and reconcile to reach at least 95% consensus. The remaining titles and abstracts will be divided to be screened by three authors and in pairs. Any disagreements will be resolved through discussion with a fourth reviewer (NS).

##### 3.1.4.4 Full-text screening

After title and abstract screening, 10% of eligible articles are screened for full-text independently by two study members (HNHL, GT), who will reconcile to reach at least 95% consensus. Next, the remaining full texts will be split among the same two team members to be screened independently and pair-wise. Any disagreements will be resolved through discussion with a third reviewer (MN).

##### 3.1.4.5 Data extraction and charting

The JBI data extraction tool for umbrella reviews is used for extraction and tabulation from the included reviews. The tool compiles general information about the studies, including study design, participant demographics (e.g., gender, age, ethnicity, health status, race, education level), search details and appraisal methods. In addition, outcomes of the reviews will be included in the form, i.e., barriers, facilitators and strategies that are reported to influence uptake of CVD risk factor screening in the review, data on screening uptake, and characteristics of individuals who do not attend screening. Data will be extracted by three study team members (HNHL, GT, MN) using a Microsoft Excel form developed by the team.

#### 3.1.5 Data analysis

A combination of thematic and framework analysis will be applied. Two members of the team will independently code the factors using open coding, discuss the codes, name and define them, before agreeing on a set of codes. This serves as a preliminary list of codes that summarises the data before the framework analysis.

After examining the actual data, the study team will discuss an appropriate behaviour change framework to use, such as the Behavioural Change Wheel (BCW) framework (34). The BCW comprises the COM-B system (i.e., the green inner core of the wheel), nine intervention functions (i.e., the red ring), and seven categories of policy (i.e., the grey outer ring). The COM-B system posits that an individual's behaviour (B) is influenced by six domains under three factors, namely capability (C), opportunity (O) and motivation (M). Capability refers to an individual's physical and psychological capabilities. Opportunity refers to an individual's environment, such as social and physical environment, that can promote or deter their behaviour. Motivation refers to cognitive and emotional processes that direct or inspire an individual to perform a behaviour. The nine intervention functions, namely education, persuasion, incentivisation, coercion, training, restriction, environmental restructuring, modelling and enablement, are strategies that can be used to promote or address any of the behavioural factors (Capability, Opportunity, Motivation) and change behaviours. They may also be used for coding as broad categories that may inform the development of strategies or interventions later on. The grey outer ring of the wheel, comprising legislation, regulations and policies, is beyond the study scope.

After an appropriate framework has been selected, a framework analysis will be carried out, informed by the relevant theoretical concepts, to generate important categories and factors that influence CVD risk factors screening as revealed by the body of evidence reviewed. The preliminary list of codes derived from the thematic round will be discussed by two coders (GT, JH) and used to iteratively develop a codebook. Subsequently, the rest of the data will be coded by three coders (HNHL, GT, MN) who will have regular discussions to adjudicate differences and refine the codebook. This process will be shared with the Project Team and Steering Group members of the project, who will offer a perspective on the dataset within the wider implementation and policy context in the Western region of Singapore.

### 3.2 Document review

#### 3.2.1 Aim

Despite a potentially significant number of internationally published articles on behavioural determinants and implementation strategies to increase CVD screening uptake, we anticipate that Singapore-specific studies are relatively few. Hence, a document review of local reports relating to CVD screening will be conducted to gather local, possibly unpublished evidence. This part of the study aims to answer the research question: How does existing international evidence align with the Singapore context?

#### 3.2.2 Data sources

Data include national or local technical manuals, evaluation reports of screening programmes, screening test review reports, population health workgroup meeting notes, and any documented learning available. Data will be provided by Project Team and Steering Group members.

#### 3.2.3 Data analysis

Inductive coding will primarily be adopted for the analysis of the document review. The documents will be divided among three study members (GT, MN, JH), who will independently review the documents and elicit three broad themes: (1) behavioural determinants for CVD screening, (2) implementation or behavioural strategies and reported effectiveness if any, and (3) characteristics of individuals who do not participate in screening. Subsequently, the members will discuss and name the codes, providing a definition for each code, agreeing on a set of codes, and formulating themes and sub-themes. This coding process will be iterative, with changes made to rename the codes and finalise the themes. The same frameworks adopted for the umbrella review may also be considered to categorise the themes. Similar to the rapid review, findings of the document review will be shared with the Project Team and Steering Group members for co-interpretation.

### 3.3 Interviews and focus groups with key stakeholders

#### 3.3.1 Aim

Qualitative data collected from key stakeholders will elicit the perspectives of professional and lay stakeholders who play critical roles in the provision, implementation and utilisation of CVD screening initiatives in the Western region of Singapore. Semi-structured interviews (35) or Focus Group Discussions (FGD) (36) will be conducted with primary care leaders and providers to gain more insight into the conceptualisation, provision and implementation of CVD screening initiatives. Interviews or FGD (depending on logistics and participant preferences) will be conducted with community-dwelling residents who are targeted by screening programmes to understand their reasons for participation or non-participation in CVD screening.

#### 3.3.2 Sampling method

##### 3.3.2.1 Primary care providers and leaders

At least two participants from private General Practitioner clinics in the Western region of Singapore, or representatives from the Ministry of Health, Health Promotion Board, or the National University Health System RHSO will be sampled. These are clinicians who are familiar with Singapore's overall screening strategy and are participating in the implementation of CVD screening programmes in Singapore.

##### 3.3.2.2 Community-dwelling residents

Residents who are 40 years old and above, eligible for a screening programme, who have or have not participated in CVD health screening before data collection, will be sampled. Participants will be recruited using purposive sampling with snowball technique based on gender, ethnicity and screening status. Sample size will be determined via thematic saturation (when fresh information can no longer be obtained from the interviews or FGD (37). Based on technical best practice recommendations for such techniques (37–39), the overall size of the relevant populations to be sampled from, and our experience with qualitative data collection on preventive health topics in Singapore, we anticipate a sample size of 20–40 participants for this part of the study.

#### 3.3.3 Data collection

The interview and FGD guides will be developed based on the COM-B model, customised according to the respective participant groups. The COM-B model is selected due to its ability to elicit factors influencing uptake of CVD screening from individual perspectives. The topic guide for residents will also include inputs from the RAC. A short socio-demographic form will be administered to capture participants’ socio-demographic information and participation in CVD screening services.

The interviews and FGD will be conducted by trained qualitative researchers face-to-face or over a video conferencing platform at a place and time convenient to the participants. The study will be explained to participants using a Study Information Sheet and consent will be obtained prior to the interview and FGD session. All interviews and FGD will be audio recorded, transcribed verbatim (translated into English when necessary) and checked for accuracy. Appendix 3 and 4 present the draft topic guides for healthcare providers and residents, respectively. Field notes and interview reflections will document any observations that occur during the interview and FGD sessions. Participants will receive a SGD20 voucher as a token of appreciation for their time and effort.

#### 3.3.4 Data analysis

Data will be transcribed by research assistants who will anonymise and de-identify participant information as needed. The study team will check each completed transcript for accuracy. All interview recordings and transcripts will be password-protected, kept securely and accessible only to study team members.

The study will apply framework analysis, which is used to generate important categories and themes that reflect behavioural determinants of CVD screening in accordance with findings from the rapid umbrella review and COM-B model. The framework analysis will follow the key steps outlined by Gale et al. (40), which provides a structured way to analyse data from various sources. Where necessary, inductive thematic coding may also be adopted.

The study adopts a hybrid inductive coding approach where the broad themes will guide the initial categorization, while codes and sub-themes are generated inductively from the data. An initial list of codes and themes derived from the rapid umbrella review will be used to guide the development of a codebook. Subsequently, the rest of the transcripts will be coded by two coders (HNHL, GT) who will discuss to reconcile the differences and refine the codebook. A matrix will be created to facilitate the comparison of codes arising from transcripts. Key themes will be generated from the codes by reviewing the matrix and making connections within and between categories, guided by the research objectives, the analytical framework and any new concepts generated inductively from the data. After the theme generation process, the team will interpret plausible meanings of the themes and further deliberate the inter-relationship among the themes.

We will apply Lincoln and Lincoln and Guba's framework (41) to assess trustworthiness across four domains: credibility, dependability, confirmability, and transferability. Credibility will be enhanced through triangulation of data sources, regular team meetings, and the use of direct participant quotations to substantiate interpretations. An analysis audit trail will also be kept to enhance dependability of the analysis, and to ensure no important data are missing from analysis and interpretation. Confirmability will be strengthened through reflexive memo-ing and collaborative coding, which helps minimize individual researcher bias. Transferability will be facilitated by providing detailed descriptions of the study context and participant characteristics, enabling readers to assess the applicability of the findings to other settings. Data analysis will be performed on QSR NVivo software v11.0.

Draft thematic structure and contents will be shared with key stakeholders such as the Steering Group, who are able to view the results within the wider practice and policy context in Singapore. Informant feedback will also be obtained by the study team as a form of member checking and further validation to the results.

## 4 Study phase 2: develop an implementation and behavioural strategy toolkit to support CVD screening

### 4.1 Strategy development process using implementation mapping

The purpose of the strategy development exercise is to map a list of behavioural determinants to a list of implementation strategies based on the findings from Phase 1. Subsequently, the strategies will be rated for their perceived feasibility and acceptability. Lastly, the shortlisted strategies that emerge higher on a perceived feasibility and acceptability matrix will be discussed with multiple stakeholders. In these discussions, more details of the strategies including their contents and mode of delivery in Singapore context will be specified. Proctor et al. (37)’s recommendations on how to specify an implementation strategy may be applied. Each strategy would be specified according to some or all seven following dimensions:

• The actor (who will carry out the strategy)
• The action (what are the actions that need to be carried out)
• Action targets (what are the action targets that the strategy is trying to impact. For example, the strategy “training” would try to impact “knowledge and skills”)
• Temporality (when the strategy should be carried out)
• Dose (how many times the strategy should be carried out)
• Implementation outcomes addressed (what are the implementation outcomes that the strategy aimed to impact)
• Theoretical justification (what is the theoretical justification for selecting the strategy)

It is anticipated that the strategies will be specified and bundled according to various settings of Singapore's healthcare system where the sequences and process flow of the strategies would be delineated. This will facilitate the process of profiling individuals who are more likely to attend screening and their counterparts. This process will be collaboratively and iteratively performed with multiple stakeholders of the study, such as the Steering Group, Project Team, primary care leaders, and RAC members. Through these co-design meetings, a set(s) of strategies that are most feasible and acceptable by consensus to various stakeholders will be selected.

Next, the content of the strategies and how they would be delivered in the local context from each group of stakeholders will be presented back to all stakeholders. They will be asked to provide their views on the final set(s) of selected strategies, potentially according to the APEASE (Acceptability, Practicability, Effectiveness/cost-effectiveness, Affordability, Safety/side-effects, Equity) criteria (42). Feedback will be collected in an open-ended format to allow participants to voice their thoughts about the strategies. If there is any strategy or implementation process that is deemed unsuitable, they will be asked to recommend other strategies or processes for improvement. Feedback received will be used to improve the strategy accordingly. These co-design meetings will be conducted by the study team who are trained in qualitative research face-to-face or over video conferencing platforms. All meetings will be audio recorded and analysed thematically for content. The outcome of the strategy development process will be a list of implementation strategies, which are promising in light of supporting evidence of effect as well as the overall implementability assessment for the context of Western Singapore.

### 4.2 Implementation research logic model (IRLM)

To make explicit the mechanisms underlying the implementation strategies leading to a set of study outcomes (as per the evidence reviewed in Phase 1, combined for local application with stakeholders’ views of Phase 2), the study will use an IRLM to articulate the determinants, determinant mechanisms and strategy mechanisms by which it is expected to achieve its desired outcomes (Appendix 5). An IRLM is a tool created to improve the specification and reproducibility of testable causal pathways involved in implementation research projects. This helps to enhance the rigour and transparency of describing complex processes involved in the adoption of evidence-based interventions in healthcare delivery systems (43). The fully specified IRLM will function as an articulated set of hypotheses for interventions (i.e., implementation strategies) and their effects—in other words, what the RHSO may expect to observe if they ultimately choose to fund and apply one or more of the identified implementation strategies to support CVD screening over a specified timeline following completion of this study. It will also function as the evaluation framework for subsequent prospective evaluation of the applied, within the area of Western Singapore, implementation strategies.

To articulate the Implementation Research Logic Model, key stakeholders will be purposively sampled from the Steering Group, Project Team, primary care leaders, and RAC members. In addition, implementation scientists, health behavioural researchers and healthcare professionals who have experienced and are familiar with the operational processes of how screenings are usually implemented in the NUHS healthcare settings may also be invited.

### 4.3 Strengths and limitations

The study will add to the global evidence of screening for CVD risk factors using data triangulated from multiple sources, collected by multiple methods, and supported by theoretical considerations. The study also attempts to contextualise international evidence to inform local evidence through active stakeholder engagement and a co-design process that fosters a sense of ownership among stakeholders. Lastly, this is one of the first studies to our knowledge in Singapore that organises a RAC and methodologically engages them to craft health screening strategies advised by the residents for the residents. This collaborative model can offer significant learning and potentially serve as a blueprint for future research aiming to involve community members in meaningful ways.

We also anticipate several limitations to the study. Firstly, the study does not offer an experimental design to perform prospective testing of the proposed implementation strategy toolkit. There can be potential selection bias for the RAC as individuals who are well-informed or have actively participated in health screening may participate instead of harder-to-reach individuals. Engaging residents and setting up the RAC may be a time-consuming process that demands extensive groundwork to foster trusting relationships within the partnership. There is also potential tension between delivering research outcomes (productivity) and active stakeholder engagement (inclusion).
