# Supplementary material for: Factors influencing cardiovascular disease screening uptake and implementation strategies to enhance cardiovascular disease screening uptake in Singapore adults: a multi-method study protocol
Source: Front Health Serv. 2026 Jan 7;5:1677666. doi: 10.3389/frhs.2025.1677666 (PMC12819692; doi:10.3389/frhs.2025.1677666)
Supplement: Supplementary file 1 [file Supplementaryfile1.pdf]

Appendix 1: Five concepts guiding the search strategy of the rapid umbrella review

	Database(s)	Concept 1 AND	Concept 2 AND	Concept 3 AND	Concept 4 AND	Concept 5
		- general population without prior diagnosis of cardiovascular disease, diabetes mellitus, hypertension or hypercholesterolemia	*- population-level health screening*	*- implementation strategies and determinants*	*- uptake or participation in screening*	*- literature reviews and meta-analyses*
**Key words (Ti/Ab)**	All	'cardiovascular*' OR 'acute coronary syndrome' OR 'coronary disease*' OR 'coronary heart disease*' OR 'coronary artery disease*' OR arteriosclerosis OR atherosclerosis OR 'myocardial ischemia' OR 'diabetes' OR hyperlipidemia OR hyperlipidaemia OR lipidemia OR lipidaemia OR 'high cholesterol' OR dyslipidemia OR dyslipidaemia OR hypercholesterolemia OR hypercholesterolaemia OR hypertriglyceridemia OR hypertriglyceridaemia OR 'high blood lipid*' OR hypertension OR 'high blood pressure'	screening OR 'early detect*' OR 'early diagnos*' OR 'health check*' OR 'healthcheck*' OR 'preventive test*' OR 'preventive care' OR 'preventive manage*' OR 'preventive effort*'	implement* OR adopt* OR strateg* OR interven* OR 'knowledge-transfer*' OR disseminat* OR diffus* OR (translat* ADJ3 / NEAR/3 knowledge*) OR barrier* OR obstruct* OR obstacle* OR facilitat* OR threat* OR hindrance* OR hinder* OR challenge* OR inhibit* OR motivat* OR promot* OR factor* OR determinant* OR predict*<br><br>**PubMed**: ("translation knowledge"(Ti/Ab:~3))	uptake OR takeup OR participat* OR comply OR compliance OR adher* OR OR attend* OR respon*	(systematic OR narrative OR scoping OR rapid OR meta-analys* OR literature OR realist OR qualitative OR search*) AND (review*)

**MeSH/Subject Headings**	PubMed	cardiovascular diseases(MeSH) OR cardiometabolic risk factor(MeSH) OR diabetes mellitus(MeSH) OR dyslipidemia(MeSH) OR hypertension(MeSH)	mass screening(MeSH) OR early diagnosis(MeSH) OR preventive medicine(MeSH)	implementation science(MeSH) OR health plan implementation(MeSH)	patient acceptance of healthcare(MeSH)	meta-analysis(Publication Type) OR meta-analysis(MeSH Terms) OR review(Publication Type)
	Embase	‘cardiovascular disease’/exp OR ‘diabetes mellitus’/exp OR ‘hyperlipidemia’/exp OR ‘dyslipidemia’/exp OR ‘hypertension’/exp OR ‘cardiovascular risk’/exp OR ‘cardiovascular risk factor’/exp	‘mass screening’/exp OR ‘early diagnosis’/exp OR ‘preventive medicine’/exp	‘implementation science’/exp OR ‘health care planning’/exp	‘patient attitude’/exp	‘meta-analysis’:it OR review:it
	PsycInfo via OVID	exp cardiovascular disorders/ OR exp diabetes/ OR exp metabolism disorders/ OR exp hypertension/ OR exp cardiovascular health/ OR exp risk factors/	exp preventive health services/ OR exp health screening/	exp health care delivery/ OR exp knowledge transfer/	exp preventive health behavior/ OR exp client participation/	meta analysis.mp,pt.OR review.pt.

Appendix 2: Eligibility criteria of the articles for the rapid umbrella review

Inclusion criteria

Condition or domain being studied

- Cardiovascular diseases will be defined as disorders of the heart and blood vessels, and include, but are not limited to, coronary heart disease, myocardial infarction and ischemic heart disease.
- Three cardiovascular diseases are selected for cardiovascular risk factors – Type 2 diabetes mellitus, hypertension and hypercholesterolemia. Diagnosis of diabetes mellitus is limited to Type 2 diabetes and does not include Type 1 diabetes, prediabetes state or gestational diabetes. Hypertension is a condition of excessively high blood pressure (140/90mmHg or higher from measurements over two days), while hypercholesterolemia is defined as raised total cholesterol (⩾ 5.0 mmol/l or 190mg/dl).
- Health screening methods should include one of the following clinical assessments:
    - Type 2 diabetes mellitus: blood sample tests after fasting to check for blood glucose levels.
    - Hypertension: measurement of blood pressure using a blood pressure monitor.
    - Hypercholesterolemia: blood sample tests after fasting to check for blood cholesterol levels.

Population

1. General population who are above 40 years of age and do not have prior diagnosis of CVD or any of the three CDs. Prior diagnosis can be described as self-reports or objective assessments in the reviews or studies.

2. Participants of the studies can also include clinicians, primary care physicians, doctors, nurses, and other allied health staff if the population who receives screening fits previous criteria.

Intervention

1. Implementation strategies aimed to increase screening uptake, adherence, or participation of population-level health screening for CVD and risk factors (i.e. at least one of the following conditions: diabetes mellitus, hypercholesterolemia, and hypertension). Strategies will need to have been tested, assessed, or evaluated.
2. Factors, determinants or predictors that influence the screening uptake, adherence, or participation.

Comparator(s)/control

Not applicable. We will include review articles consisting of studies without control/comparator groups.

Outcome

Data on uptake, adherence, or participation rates of screening for CVD and risk factors.

Types of studies

Review articles such as scoping, rapid, realist, systematic reviews, meta-analysis and other types of literature reviews that describe the review methodology, including a search strategy and the databases searched.

Exclusion criteria

Article(s) will be excluded if it:

- Does not include the targeted population (which is defined as individuals above 40 years old without prior diagnosis of CVD or the three CVDs) in population who receives screening.
- Does not include screening using any clinical assessment specified above (i.e. blood sample tests for blood sugar and cholesterol levels, and measurement of blood pressure).
- Only evaluates effectiveness of screening and does not have any data on either the implementation strategies or the determinants that affect screening uptake.
- Does not have any data on the screening uptake.

Appendix 3: Qualitative topic guide for primary care leaders and providers

Introduction

1. Could you tell me more about your clinic?

   Prompt:

   a. Does your clinic join HealthierSG (HSG)?

   b. Is this clinic part of Primary Care Network (PCN)?

   c. How long have you been working in this clinic?

2. Could you describe a typical day at work?

   Prompt:

   a. How many patients do you see in a day?

   b. What is the catchment area of your patients?

   c. What is the proportion of your patients who have DHL and no DHL?

Experience with DHL screening

1. How do you conduct DHL screening in your clinic?

   Prompt:

   a. What is a typical screening process involved? Who does what and how?

2. What are other screening initiatives for DHL that you/your clinic participate in? Example: Screen-for-life, Community-based screening, Workplace screening

   If they participate, ask:

3. How is DHL screening conducted in these initiatives?

   Prompt:

   a. What is a typical screening process involved? Who does what and how?

If they do not participate, skip to the next segment.

Barriers and facilitators in providing DHL screening

4. As a provider, what do you think are key enablers that make DHL screening work well in primary care setting?

   Prompt:

   a. At provider level: presence of knowledge and skills, beliefs about screening outcomes, roles, capability and resources
   b. At system level: policy mandate, incentive
   a. At resident level: relationship between residents and GPs

5. What are key barriers you face when conducting DHL screening in your clinic?

   Prompt:

   b. At provider level: time constraints, manpower limitations, lack of IT capability, data management, invitation delivery, pathway to referral or follow up
   c. At system level: cost/ funding, insurance coverage, lack of incentive, other systemic constraints
   d. At resident level: lack of knowledge, awareness, motivation, social support, accessibility (cost, timing, location)

6. Have you tried to work around these limitations? How did the efforts go?

Profiles of screening and non-screening residents

7. Could you describe the profile of residents who usually attend screening in your clinic, or in the above screening initiatives?

   Prompt: What are some demographic factors that describe them?

   - Age

- Gender
- Socio-economic status (SES)
- Educational status
- Family history
- Smoking status

8. Conversely, could you describe the profile of residents who usually do not attend screening?

   Prompt: What are some demographic factors that describe them?

   - Age
   - Gender
   - Socio-economic status (SES)
   - Educational status
   - Family history
   - Smoking status

9. In your opinion, why don't they go for screening?

   Prompt:

   a. Capability: How aware are the residents about screening and benefits of screening?
   b. Opportunity: How accessible is your clinic in terms of opening hours, location, availability of staff?
   c. Motivation: What are their beliefs about risks of DHL or benefits of screening?

Strategies to increase DHL screening in primary care setting

10. From a provider perspective, what would encourage you or fellow GPs to provide more DHL screening in primary care?

Prompt:

a. What changes can be made to the existing screening process/ system?

b. What changes can be made to motivate more residents to attend screening?

11. How might the strategies you suggested address the barriers you mentioned earlier?

12. How can these strategies be carried out?

Prompt:

a. Who should be carrying out the strategy? What does this person/people do?

b. For whom does this strategy work?

c. What does this strategy entail? (e.g. the content, structure, format)

If time allows, discuss the top three or five strategies nominated by the RHSO stakeholders and GP panel

a. ***Reallocate responsibilities*** *related to screening to different care personnel (task-shifting)*

b. Create and use ***reminders for doctors*** *to offer residents for screening (physician reminders)*

c. ***Display ads in clinics and public spaces*** *to raise awareness about screening*

13. On a scale from 1 to 5, 1 being extremely unfeasible and 5 being extremely feasible, how would you rate the feasibility of these strategies? Why do you rate them as such?

14. On a scale from 1 to 5, 1 being extremely unacceptable and 5 being extremely acceptable, how would you rate the acceptability of these strategies? Why do you rate them as such?

15. For each of these strategies, could you think of:

    a. Who should be carrying out the strategy? What does this person/people do?

    b. For whom does this strategy work?

    c. What does this strategy entail? (e.g. the content, structure, format)

Thank you for taking the time to have this discussion today.

Appendix 4: Qualitative topic guide for residents

Introduction

1. Reiterate the objectives and context as reminder
2. Self-introduction within the group

Screener

1. Why did you decide to go for screening?
    a. Do your family or friends attend CVD screening? How does their decision to go (or not to go) affect you?
    b. How do you view your current lifestyle and cardiovascular health? How important is health screening for you?
    c. What are the pros and cons of screening? Does your knowledge of the benefits motivate you to go for screening? Why or why not?
    d. What are, if any, your concerns about CVD screening?
        i. Prompts: cost, physical access (time, location, appointment times)

2. What would encourage you to sign up for CVD screening? What would encourage you to attend screening?
    a. What changes can be made to the existing screening programmes to/How might the strategies you suggested address the concerns you mentioned earlier that prevent you from attending screenings?
    b. How can these strategies reach you effectively? (Prompts about role of implementor, mode of reach, format of strategy, strategy distribution timing and frequency, etc)

    c. *if time allows, discuss the top three or five strategies nominated by the RACs, the GP panel and RHSO stakeholders.

Non-screener

16. What do you know about CVD screening programmes in Singapore? (different settings e.g. HealthierSG, Screen For Life, community screening, etc)
    a. What do you know about what is done during screening events?

17. Why don't you go for screening?
    a. Do your family or friends attend CVD screening? How does their decision to go (or not to go) affect you?
    b. How do you view your current lifestyle and cardiovascular health? How important is health screening for you?
    c. What are the pros and cons of screening? Does your knowledge of the benefits motivate you to go for screening? Why or why not?
    d. What are your concerns about CVD screening?
        i. Prompts: cost, physical access (time, location, appointment times)
    e. Have you had previous negative experience with health services that might have affected your decision to attend screenings? Can you describe the experience?

18. What would encourage you to sign up for CVD screening? What would encourage you to attend screening?

a. What changes can be made to the existing screening programmes to/How might the strategies you suggested address the concerns you mentioned earlier that prevent you from attending screenings?
b. How can these strategies reach you effectively? (Prompts about role of implementor, mode of reach, format of strategy, strategy distribution timing and frequency, etc)
c. *if time allows, discuss the top three or five strategies nominated by the RACs, the GP panel and RHSO stakeholders.

Thank you for taking the time to have this discussion today and I hope that everyone has had a fruitful experience.

Appendix 5: The Implementation Research Logic Model (IRLM)

**Implementation Determinants**		**Implementation strategies**	**Mechanisms**	**Outcomes**	
Intervention characteristics				Implementation	
Inner setting					
Outer setting				Service	
Characteristics of individuals					
Process		**Clinical intervention**		Patient/client	
